# Supplementary material for: Tabular diffusion counterfactual explanations
Source: Front Artif Intell. 2026 Feb 13;9:1743495. doi: 10.3389/frai.2026.1743495 (PMC12946002; doi:10.3389/frai.2026.1743495)
Supplement: Supplementary file 1 [file Data_Sheet_1.pdf]

Supplementary Material

1 PROOF OF THE CLOSENESS OF THE APPROXIMATED GUMBEL SOFTMAX DISTRIBUTION

LEMMA 1.1. *Given $\pi, \widetilde{x} \in \triangle^{K-1}$ be a probability simplex vector and $\widetilde{x} \geq \widetilde{x}_{min} > 0$. For an arbitrary temperature $\tau \in \mathbb{R}^+$, the lower bound of*

$$l = \min \sum_i^K \frac{\pi_i}{\widetilde{x}_i^\tau} \tag{S1}$$

has the range

$$1 \leq l \leq K^\tau \tag{S2}$$

The upper bound is:

$$\sum_i^K \frac{\pi_i}{\widetilde{x}_i^\tau} \leq \frac{1}{\widetilde{x}_{min}^\tau} \tag{S3}$$

PROOF. We know that $f(u) = u^\tau$ for $\tau > 0$ is strictly convex. Thus, we know that $\sum_i^K \frac{\pi_i}{\widetilde{x}_i^\tau}$ is also strictly convex, indicating that any minimum point is the global minimum. Then, we can set up the constrained optimization problem:

$$\min_{x \in \Delta^{K-1}} F(\widetilde{x}) = \sum_i^K \pi_i \widetilde{x}_i^{-\tau} \tag{S4}$$

$$\text{subject} \quad \sum_j^K \widetilde{x}_j = 1 \tag{S5}$$

The Lagrangian multiplier can be established as:

$$\mathcal{L}(\widetilde{x}, \lambda) = \sum_i^K \pi_i \widetilde{x}_i^{-\tau} + \lambda(\sum_j^K \widetilde{x}_j - 1) \tag{S6}$$

Taking the derivative w.r.t. $\widetilde{x}_j$:

$$\frac{\partial \mathcal{L}}{\partial \widetilde{x}_j} = \pi_j \widetilde{x}_j^{-\tau-1}(-\tau) + \lambda = 0 \quad \rightarrow \quad \widetilde{x}_j = (\frac{\tau \pi_j}{\lambda})^{\frac{1}{\tau+1}} \tag{S7}$$

Enforcing the constraint $\sum_i^K \widetilde{x}_i = 1$ leads to:

$$\lambda = \tau(\sum_i^K \pi_i^{\frac{1}{\tau+1}})^{\tau+1} \tag{S8}$$

Combining together:

$$\widetilde{x}_i^* = \frac{\pi_i^{\frac{1}{\tau+1}}}{\sum_j^K \pi_j^{\frac{1}{\tau+1}}} \tag{S9}$$

The minimum value of $F(\widetilde{x})$ is:

$$F(\widetilde{x}^*) = (\sum_i^K \pi_i^{\frac{1}{\tau+1}})^{\tau+1} \tag{S10}$$

By generalized Hölder inequality, this has the range:

$$1 \leq F(\widetilde{x}^*) \leq K^{\tau} \tag{S11}$$

The upper bound can be derived by setting $\widetilde{x}_i = \widetilde{x}_{min}$. Then, the upper bound becomes:

$$\sum_i^K \frac{\pi_i}{\widetilde{x}_i^{\tau}} \leq \frac{1}{\widetilde{x}_{min}^{\tau}} \sum_{i=1}^K \pi_i = \frac{1}{\widetilde{x}_{min}^{\tau}} \tag{S12}$$

THEOREM 1.2. *vLet $\widetilde{x}, \pi \in \Delta^{K-1}$ and the temperature $\tau \in \mathbb{R}^+$. Define $\widetilde{x}_{min}$ the minimum value $\widetilde{x}$ can take. The KL divergence between p_{GS} defined in Equation* **??** *and its approximation p_θ in Equation* **??** *is bounded as follows:*

$$
\begin{aligned}
\mathrm{KL}(p_{GS}\|p_\theta) < &- K(\tau+1)\log\widetilde{x}_{min} + (K-1)\log\tau + (K-1)\log[1-\widetilde{x}_{min}] \\
&+ \log\Gamma(K) + K\log[(1-\widetilde{x}_{min})/(K-1)!] \\
\mathrm{KL}(p_{GS}\|p_\theta) > &\, K\tau\log\widetilde{x}_{min} + (K-1)\log\tau + (K-1)\log[\widetilde{x}_{min}] + \log\Gamma(K) \\
&+ K\log[\widetilde{x}_{min}/(K-1)!]
\end{aligned}
$$

PROOF. We want to bound the KL divergence between

$$p_\theta(\widetilde{x}|\pi) = \frac{1}{Z(\pi)}\prod_i^K \pi^{\widetilde{x}_i} \tag{S13}$$

and

$$p(\widetilde{x}|\pi,\tau) = \Gamma(K)\tau^{K-1}\left(\sum_i^K \frac{\pi_i}{\widetilde{x}_i^\tau}\right)^{-K}\prod_i^K \frac{\pi_i}{\widetilde{x}_i^{\tau+1}} \tag{S14}$$

where K is the number of classes for the categorical variable and $\pi, \widetilde{x} \in \triangle^{K-1}$.

1.0.1 Derivation of the upper bound

$$\mathrm{KL}(p(\widetilde{x}|\pi,\tau)\|p_\theta(\widetilde{x}|\pi)) = \mathbb{E}\left[\log\left[\Gamma(K)\tau^{K-1}\prod_{i=1}^{K}\frac{Z(\pi)\pi_i^{1-\widetilde{x}_i}\widetilde{x}_i^{-\tau-1}}{\sum_j^K \pi_j\widetilde{x}_j^{-\tau}}\right]\right] \tag{S15}$$

$$= \mathbb{E}\left[\log\Gamma(K) + (K-1)\log\tau + \sum_i^K \log\frac{Z(\pi)\pi_i^{1-\widetilde{x}_i}\widetilde{x}_i^{-\tau-1}}{\sum_j^K \pi_j\widetilde{x}_j^{-\tau}}\right] \tag{S16}$$

$$\leq \mathbb{E}\left[(K-1)\log\tau + \sum_i^K \log\frac{Z(\pi)(1-\widetilde{x}_{min})^{1-\widetilde{x}_i}\widetilde{x}_i^{-\tau-1}}{\sum_j^K \pi_j\widetilde{x}_j^{-\tau}}\right] + \log\Gamma(K)$$

$$\text{by}\quad \pi_i^{1-\widetilde{x}_i} \leq (1-\widetilde{x}_{min})^{1-\widetilde{x}_i} \tag{S17}$$

$$\leq \mathbb{E}\left[\sum_i^K \log\frac{[\max_{1\leq j\leq K}\pi_j](1-\widetilde{x}_{min})^{1-\widetilde{x}_i}\widetilde{x}_i^{-\tau-1}}{(K-1)!\sum_j^K \pi_j\widetilde{x}_j^{-\tau}}\right] + (K-1)\log\tau$$

$$+\log\Gamma(K)\quad \text{Lebesgue measure of } \Delta^{K-1} \tag{S18}$$

$$\leq \mathbb{E}\left[\sum_i^K \log\frac{[\max_{1\leq j\leq K}\pi_j](1-\widetilde{x}_{min})^{1-\widetilde{x}_i}\widetilde{x}_i^{-\tau-1}}{(K-1)!}\right] + (K-1)\log\tau + \log\Gamma(K)$$

$$\text{by Lemma } 1.1 \tag{S19}$$

$$= \mathbb{E}\left[\sum_i^K \log\widetilde{x}_i^{-\tau-1}\right] + (K+1)\log\tau + \log\Gamma(K) + (K-1)\log(1-\widetilde{x}_{min})$$

$$+ K\log\frac{1-\widetilde{x}_{min}}{(K-1)!} \tag{S20}$$

$$= \mathbb{E}\left[\sum_i^K (-\tau-1)\log\widetilde{x}_i\right] + (K-1)\log\tau + \log\Gamma(K)$$

$$+ (K-1)\log(1-\widetilde{x}_{min}) + K\log\frac{1-\widetilde{x}_{min}}{(K-1)!} \tag{S21}$$

$$< -K(\tau+1)\log(1-\widetilde{x}_{min}) + (K-1)\log\tau + \log\Gamma(K)$$

$$+ (K-1)\log(1-\widetilde{x}_{min}) + K\log\frac{1-\widetilde{x}_{min}}{(K-1)!} \tag{S22}$$

1.0.2 Derivation of the lower bound

$$\mathrm{KL}(p(\widetilde{x}|\pi,\tau)\|p_\theta(\widetilde{x}|\pi)) = \mathbb{E}\left[\log\left[\Gamma(K)\tau^{K-1}\prod_{i=1}^{K}\frac{Z(\pi)\pi_i^{1-\widetilde{x}_i}\widetilde{x}_i^{-\tau-1}}{\sum_j^K \pi_j \widetilde{x}_j^{-\tau}}\right]\right] \tag{S23}$$

$$= \mathbb{E}\left[\log\Gamma(K) + (K-1)\log\tau + \sum_i^K \log\frac{Z(\pi)\pi_i^{1-\widetilde{x}_i}\widetilde{x}_i^{-\tau-1}}{\sum_j^K \pi_j \widetilde{x}_j^{-\tau}}\right] \tag{S24}$$

$$\geq \mathbb{E}\left[\log\Gamma(K) + (K-1)\log\tau + \sum_i^K \log\frac{Z(\pi)\pi_i^{1-\widetilde{x}_i}\widetilde{x}_i^{-\tau-1}}{\widetilde{x}_{min}^{-\tau}}\right]$$

$$\text{by Lemma } 1.1 \tag{S25}$$

$$= \mathbb{E}\left[(K-1)\log\tau + \sum_i^K \log\frac{Z(\pi)\pi_i^{1-\widetilde{x}_i}\widetilde{x}_i^{-\tau-1}}{\widetilde{x}_{min}^{-\tau}}\right] + \log\Gamma(K) \tag{S26}$$

$$> \mathbb{E}\left[(K-1)\log\tau + \sum_i^K \log\frac{\widetilde{x}_{min}\pi_i^{1-\widetilde{x}_i}\widetilde{x}_i^{-\tau-1}}{(K-1)!\widetilde{x}_{min}^{-\tau}}\right] + \log\Gamma(K)$$

$$\text{by Lebesgue measure of } \Delta^{K-1} \tag{S27}$$

$$> \mathbb{E}\left[(K-1)\log\tau + \sum_i^K (-\tau-1)(\log\widetilde{x}_i - \log\widetilde{x}_{min})\right] + \log\Gamma(K)$$

$$+ K\log\frac{1}{(K-1)!} + (K-1)\log\widetilde{x}_{min} \tag{S28}$$

$$= \mathbb{E}\left[(K-1)\log\tau + \sum_i^K (\tau+1)(\log\widetilde{x}_{min} - \log\widetilde{x}_i)\right] + \log\Gamma(K)$$

$$+ K\log\frac{1}{(K-1)!} + (K-1)\log\widetilde{x}_{min} \tag{S29}$$

$$> (K-1)\log\tau + K(\tau+1)\log\widetilde{x}_{min} + \log\Gamma(K)$$

$$+ K\log\frac{1}{(K-1)!} + (K-1)\log\widetilde{x}_{min} \tag{S30}$$
